# Penicilliumin B, a novel sesquiterpene methylcyclopentenedione from a deep sea-derived *Penicillium* strain with renoprotective activities

**DOI:** 10.1038/s41598-017-11007-4

**Published:** 2017-09-07

**Authors:** Xiuping Lin, Qinyu Wu, Yuying Yu, Zhi Liang, Yonghong Liu, Lili Zhou, Lan Tang, Xuefeng Zhou

**Affiliations:** 1 Chinese Academy of Sciences (CAS) Key Laboratory of Tropical Marine Bio-resources and Ecology, Guangdong Key Laboratory of Marine Materia Medica, South China Sea Institute of Oceanology, CAS, Guangzhou, 510301 China; 2 Biopharmaceutics, Guangdong Provincial Key Laboratory of New Drug Screening, School of Pharmaceutical Sciences, Southern Medical University, Guangzhou, 510515 China; 3 State Key Laboratory of Organ Failure Research, National Clinical Research Center of Kidney Disease, Division of Nephrology, Nanfang Hospital, Southern Medical University, Guangzhou, 510515 China

## Abstract

A novel sesquiterpene methylcyclopentenedione, penicilliumin B (**1**), was obtained from a deep sea-derived fungus *Penicillium* sp. F00120, together with three known sesquiterpene cyclohexenones (**2**–**4**). Penicilliumin B (**1**), presenting the first example with the sesquiterpene cyclopentenedione skeleton as natural products, was structurally determined by analysis of the NMR and MS spectroscopic data, while the absolute configurations were assigned by single-crystal X-ray experiments. The plausible biosynthetic pathway of the unusual cyclopentenone skeleton of **1** was proposed. Penicilliumin B (**1**), with low toxicity, was showed significant potential to inhibit the kidney fibrogenic action *in vitro*, by a mechanism dependent on disruption of oxidative stress, presenting a new type of promising renoprotective agent.

## Introduction

Sesquiterpene quinones/hydroquinones (SQ/SHQ) are common in marine sponges^1^, seaweed^2^, and marine or terrestrial microorganisms as well^3, 4^. SQ/SHQ are attractive because of their widely various biological activities, including antitumor^1^, antivirus^5, 6^, antimicrobial^7, 8^, and NF-κB inhibitory activities^9^. Several sponge-derived SQ or SHQ, such as avarol, avarone, illimaquinone, and bolinaquinone, offer promising opportunities for new antitumor agents^1^. Sesquiterpene cyclohexenones, structurally closely related to SQ, are relative rare in nature. Most of the cyclohexenone derivatives, obtained from plant-associated and marine-derived fungi, exhibited *in vitro* antitumor^10–12^ or antimicrobial activities^13, 14^.

Marine-derived fungi have shown promising potential to produce diverse bioactive metabolites with novel skeletons. During our continuous chemical study of marine-derived fungi, a drimane sesquiterpene cyclohexenone, penicilliumin A (**2**, also named as purpurogemutantidin)^12^, was obtained from fungus *Penicillium* sp. F00120 isolated from a deep sea sediment sample in 2012^11^. Recently, in order to discover more unique and bioactive compounds from this *Penicillium* strain, the fermentation condition was optimized and a novel sesquiterpene cyclopentenone named penicilliumin B (**1**), represents an unusual drimane sesquiterpene methylcyclopentenedione carbon skeleton, was isolated from the cultures, together with three known cyclohexenone derivatives, penicilliumin A (**2**), macrophorin A (**3**) and purpurogemutantin (**4**). Herein, the isolation, structural determination, plausible bioconversion route, and bioactivity evaluation of these compounds were reported.

## Results and Discussion

**Figure 1:**
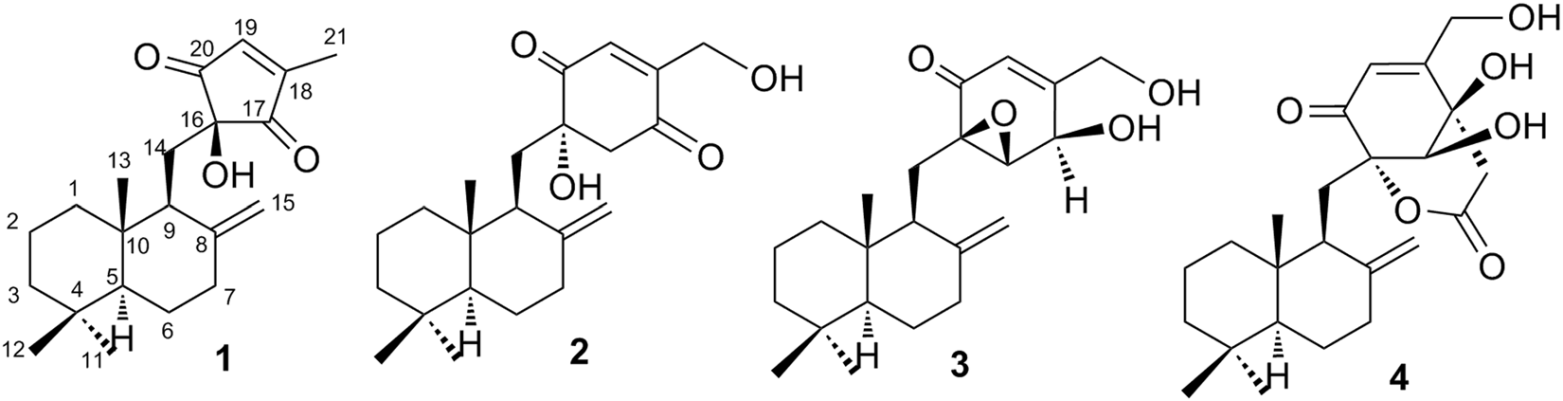
Structures of **1**–**4**.

The strain of *Penicillium* sp. F00120 was fermented on a solid rice substrate culture. The EtOAc extract of its solid culture was subjected to silica gel and Sephadex LH-20 column chromatography (CC). Compounds **1**–**4** (Fig. 1) were isolated and purified by semipreparative HPLC finally. The structures of known compounds penicilliumin A (**2**)^11, 12^, macrophorin A (**3**), and purpurogemutantin (**4**)^12^, were determined by NMR data analysis and comparison with the literature data.

**Figure 2:**
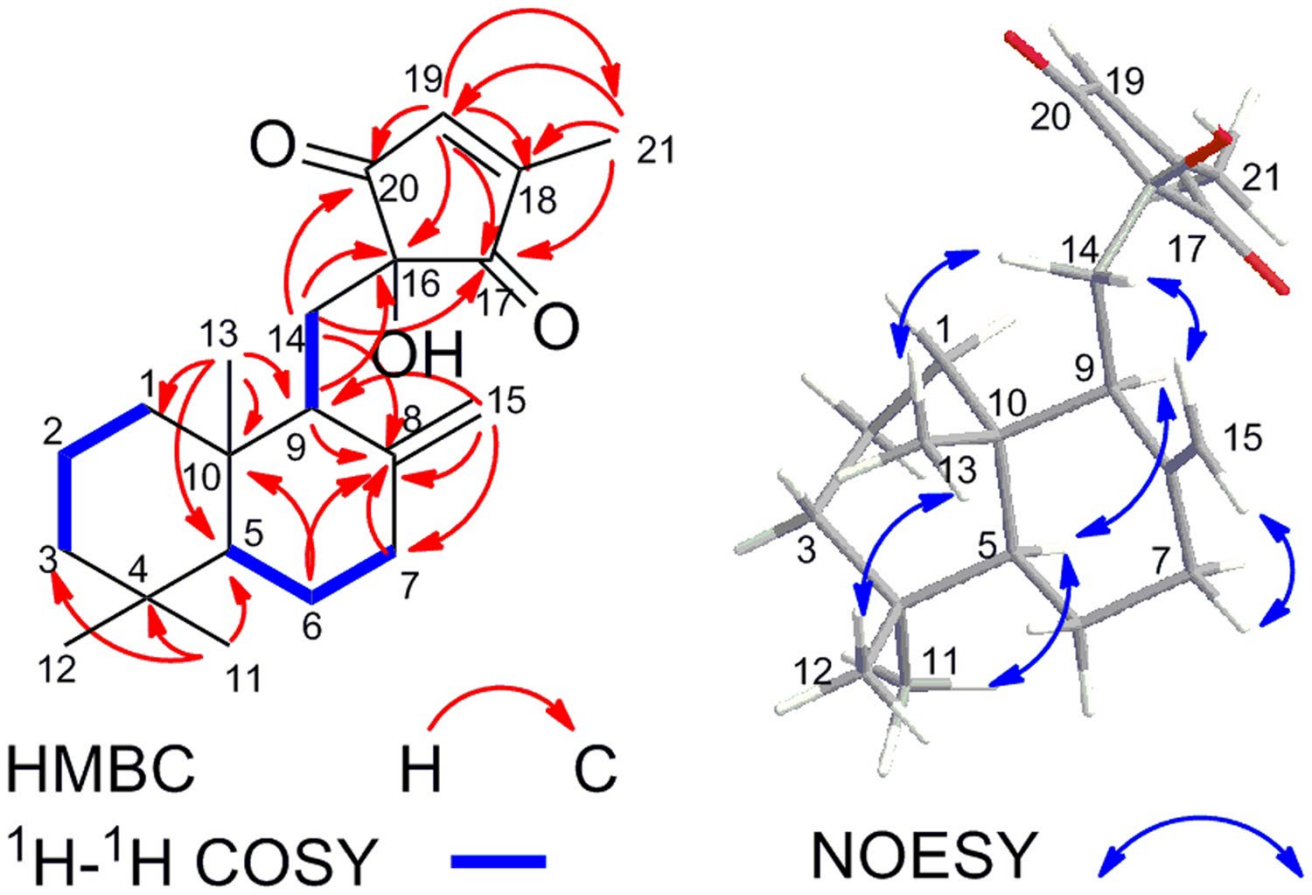
Key HMBC^1^, H-^1^H COSY and NOESY correlations of **1**.

Penicilliumin B (**1**) was obtained as colorless crystals. Its molecular formula, C_21_H_30_O_3_, was determined by positive HR-ESI-MS (*m/z* 353.2114 [M + Na]^+^, calcd for C_21_H_30_O_3_Na, 353.2087; *m/z* 331.2267 [M + H]^+^, calcd for C_21_H_31_O_3_, 331.2268), corresponding to seven degrees of unsaturation. The^1^H and^13^C NMR data of **1** (Table 1, Supporting Information, Figures S10−S12) were closely similar to those of penicilliumin A (**2**)^11^, except that the CH_2_ and the CH_2_OH signals in cyclohexenone moiety of **2** were replaced by an exocyclic CH_3_ signal. The same drimane sesquiterpene moiety of **1** with **2** was also confirmed by HSQC, HMBC and ^1^H-^1^H COSY correlations (Fig. 2, Figures S13−S15). The key HMBC correlations of H_2_-14 (*δ*_H_ 1.88, 2.02) with C-16 (*δ*_C_ 75.5), C-17 (*δ*_C_ 203.2) and C-20 (*δ*_C_ 201.6), H-19 (*δ*_H_ 6.92) with C-16, C-17, C-18 (*δ*_C_ 160.4), C-20 and C-21 (*δ*_C_ 11.9), H-21 (*δ*_H_ 2.07) with C-17, C-18, and C-19 (*δ*_C_ 142.8), suggested **1** contained a 4-methyl-4-cyclopentene-1,3-dione fragment connecting with C-14 (*δ*_C_ 29.9), instead of quinone or hexatomic moiety (Fig. 2). Thus, the whole planar structure of **1** was elucidated.

**Table 1:** NMR Spectroscopic Data (500 MHz, CDCl_3_) for Penicilliumin B (**1**).

position	δ_H_ (*J* in Hz)	δ_C_ (type)	position	δ_H_ (*J* in Hz)	δ_C_ (type)
1	0.95 (dt, 12.4, 4.2) 1.60 (br d, 12.4)	38.5 (CH_2_)	12	0.74 (s)	21.5 (CH_3_)
2	1.47 (m) 1.50 (m)	19.2 (CH_2_)	13	0.59 (s)	14.6 (CH_3_)
3	1.16 (dt, 12.9, 4.5) 1.35 (br d, 12.9)	41.8 (CH_2_)	14	1.88* 2.02 (dd, 14.5, 10.5)	29.9 (CH_2_)
4	/	33.6 (C)	15	4.52 (br s), 4.84 (br s)	108.3 (CH_2_)
5	1.10 (dd, 12.6, 2.6)	55.3 (CH)	16	/	75.5 (C)
6	1.26 (m) 1.72 (m)	24.3 (CH_2_)	17	/	203.2 (C)
7	1.91* 2.32 (ddd, 11.5, 3.7, 2.5)	37.9 (CH_2_)	18	/	160.4 (C)
8	/	149.4 (C)	19	6.92 (s)	142.8 (CH)
9	1.89*	51.3 (CH)	20	/	201.6 (C)
10	/	40.0 (C)	21	2.07 (s)	11.9 (CH_3_)
11	0.84 (s)	33.4 (CH_3_)			

^*^Overlap.

**Figure 3:**
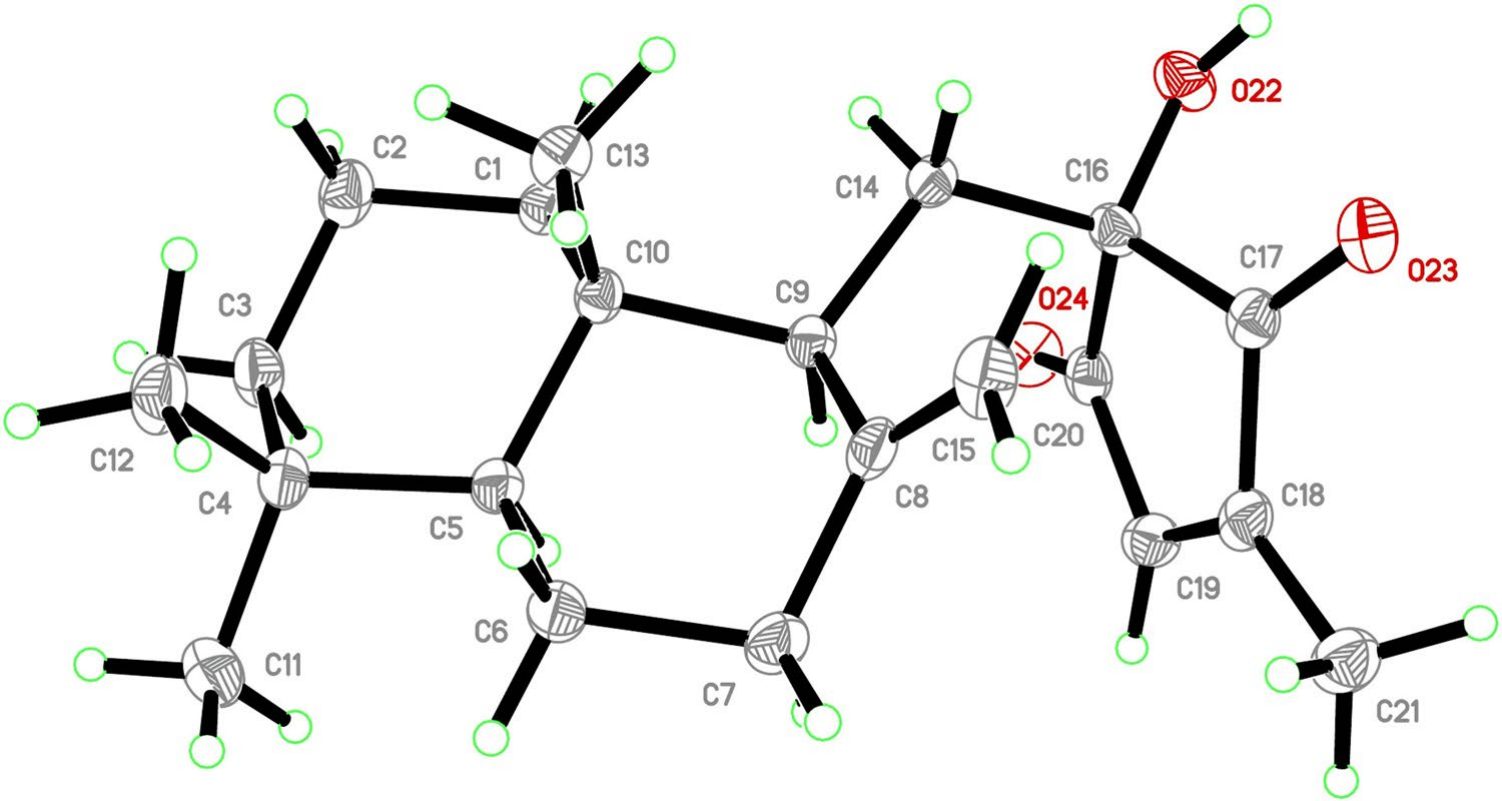
X-ray crystallographic structure of **1**.

The relative configurations were discussed and assigned by NOESY spectrum, especially the observed correlations of H_3_-11/H-5/H-9 and H_3_-12/H_3_-13/H_2_-14 (Fig. 2, Figure S16). A single crystal of **1** was obtained by recrystallization from acetone/methanol system. The structure and absolute configuration of **1** was confirmed by single-crystal X-ray diffraction analysis with Cu K*α* radiation (Fig. 3). The Flack parameter, 0.02(7), permitted the establishment of the absolute configuration of **1** ^15^. Therefore, the overall configuration of **1** was assigned as 5 *S*, 9 *S*, 10 *S*, 16 *S*, and named as penicilliumin B.

**Figure 4:**
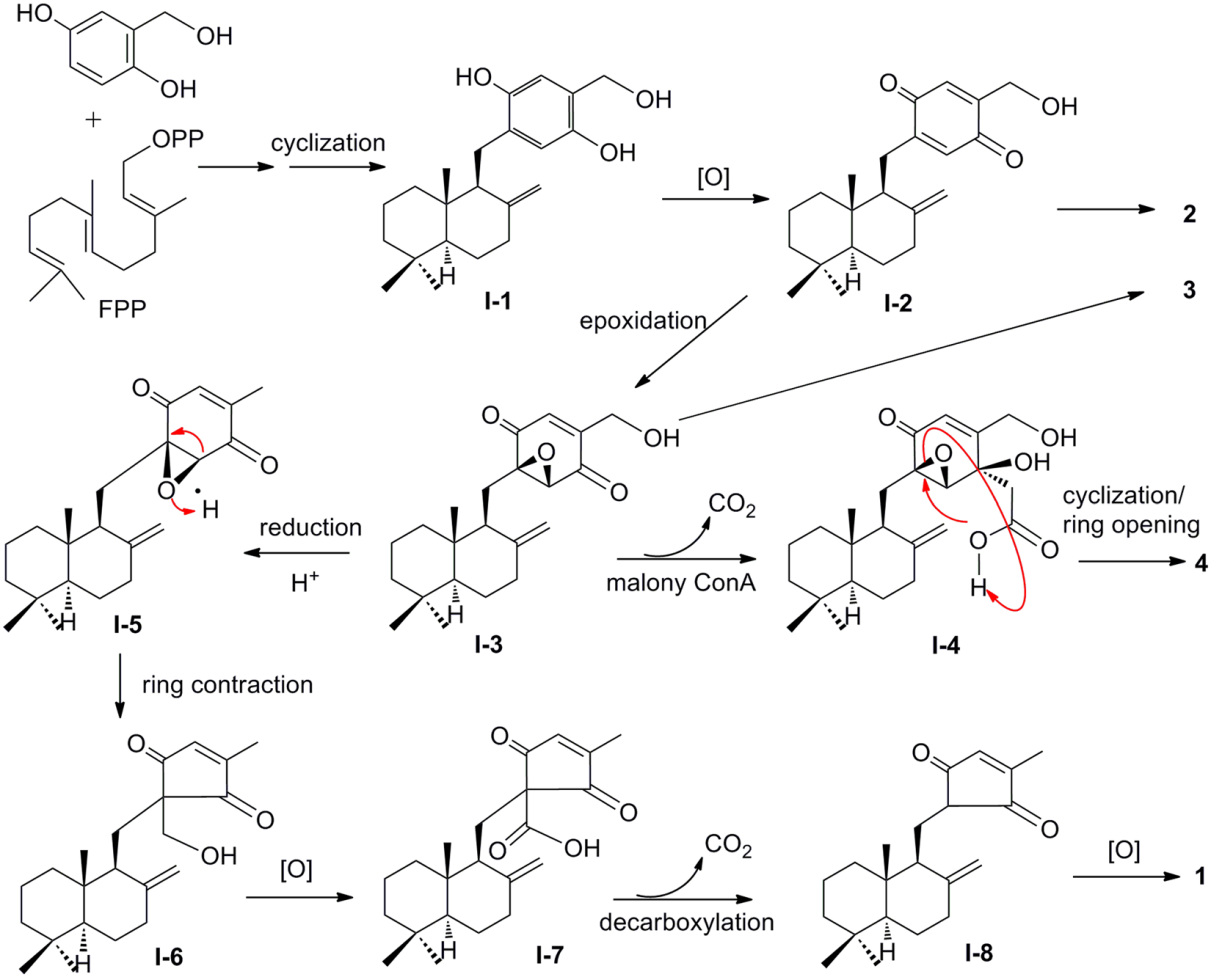
Plausible Biosynthetic Pathway of Compounds **1**−**4**.

A proposed biogenetic pathway for the assembly of **1**−**4** in this fungus was shown in Fig. 4, and the unusual sesquiterpene methylcyclopentenedione was supposed to be derived from the usual SQ. In brief, the cyclopentenedione moiety of **1** was biosynthesized from epoxy cyclohexenone of **I-3**, by ring contraction, oxidation, and decarboxylation of *β*-keto acids mainly. It is interesting to note that, decarboxylic reaction might also happened in malonylation of **I**-**3** to give sesquiterpene cyclohexenone **4**. Natural cyclopentenediones is rare, and **1** presents the first example with the structure of sesquiterpene cyclopentenedione as natural products.

Sesquiterpene cyclohexenones **2**–**4** were reported with their significant or moderate inhibitory activities against several cancer cells^11, 12^. However, our bioactive assay showed sesquiterpene methylcyclopentenedione **1** has nearly no inhibitory activities against eight cancer cells (Supporting Information, Table S1). Meanwhile, **1** was also inactive in all of the antibacterial (*Escherichia coli*, *Salmonella enterica*, *Staphylococcus aureus*), antituberculosis (*M. tuberculosis* H37Ra), antiviral (H1N1, H3N2, EV71), and anti-inflammatory (COX-1, COX-2, NF-κB luciferase) assays (Supporting Information, Tables S2−S4 and Figure S1), much different with SQ or sesquiterpene cyclohexenones derivatives, which are owing broad spectrum of biological activities including those above^5–14^.

**Figure 5:**
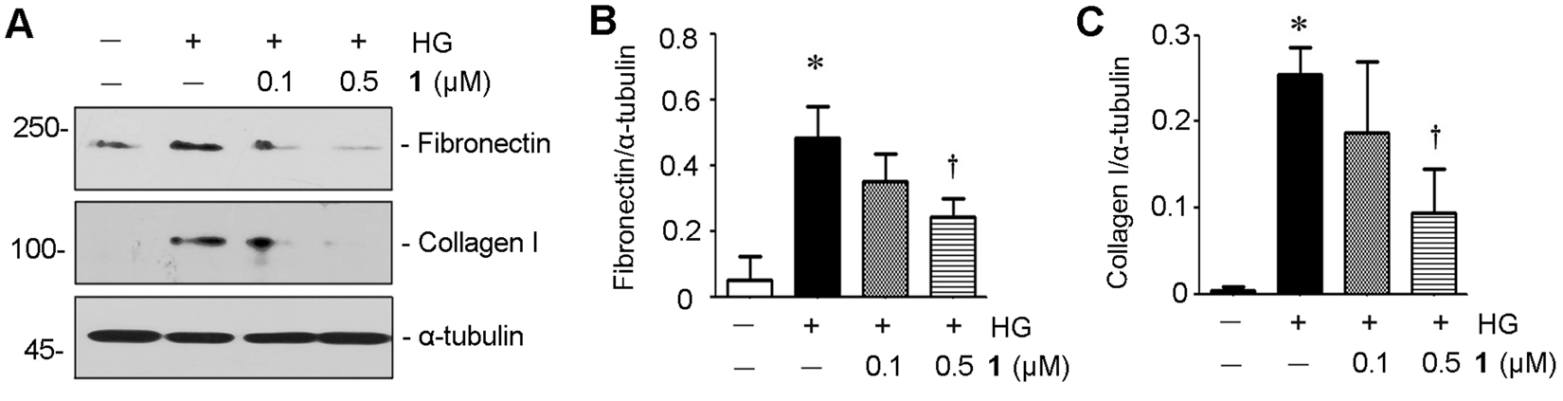
Penicilliumin B (**1**) inhibits fibrogenic action of high glucose in RMC cells. RMC cells were incubated with D-glucose (25 mM) for 48 h in the absence or presence of different concentrations (0.1, 0.5 μM) of **1**. Cell lysates after various treatments as indicated were immunoblotted with antibodies against fibronectin, collagen I and α-tubulin (**A**). Full-length blots are presented in Supporting Information Figure S2. Graphic presentations of fibronectin (**B**) and collagen I (**C**) protein expressions in four groups as indicated. *p < 0.05 vs control (n = 3). †*p* < 0.05 vs HG alone (n = 3).

During our studies of screening for renoprotective agent in marine natural products, penicilliumin B (**1**) was showed promising potential to inhibit the kidney fibrogenic action *in vitro*. The results revealed that, **1** with 0.5 μM could significantly inhibit the fibrogenic action of high glucose (HG) in rat glomerular mesangial cells (RMC), as detected by Western blotting analysis of fibronection and collagen I (Fig. 5), whose production is a main hallmark of renal fibrosis^16, 17^.

**Figure 6:**
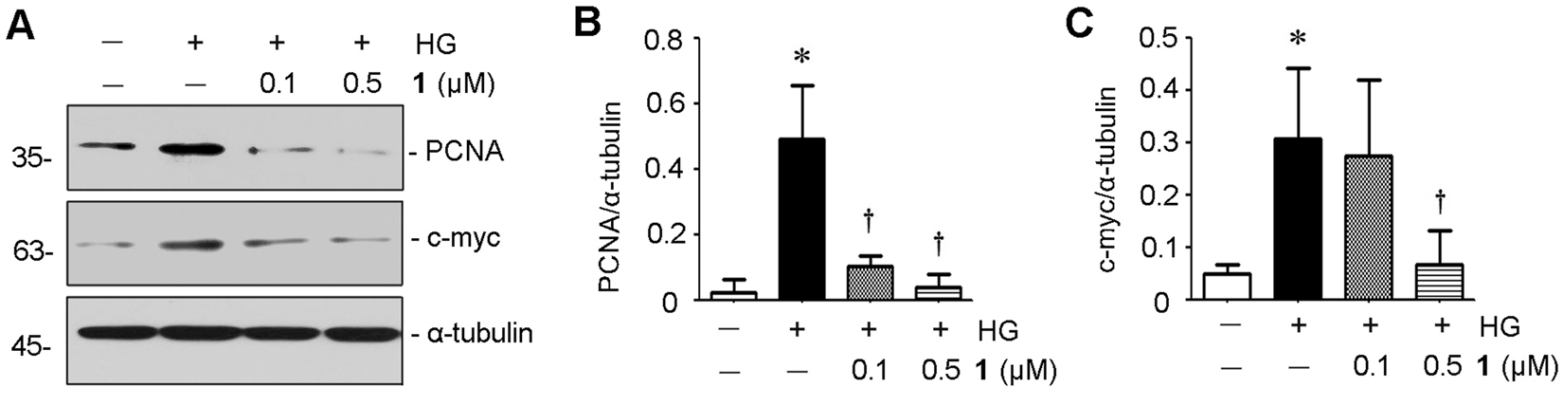
Penicilliumin B (**1**) inhibits proliferative action of high glucose in RMC cells. RMC cells were incubated with D-glucose (25 mM) for 48 h in the absence or presence of different concentrations (0.1, 0.5 μM) of **1**. Cell lysates after various treatments as indicated were immunoblotted with antibodies against PCNA, c-myc and α-tubulin (**A**). Full-length blots are presented in Supporting Information Figure S3. Graphic presentations of PCNA (**B**) and c-myc (**C**) protein expressions in four groups as indicated. *p < 0.05 vs control (n = 3). †*p < *0.05 vs HG alone (n = 3).

**Figure 7:**
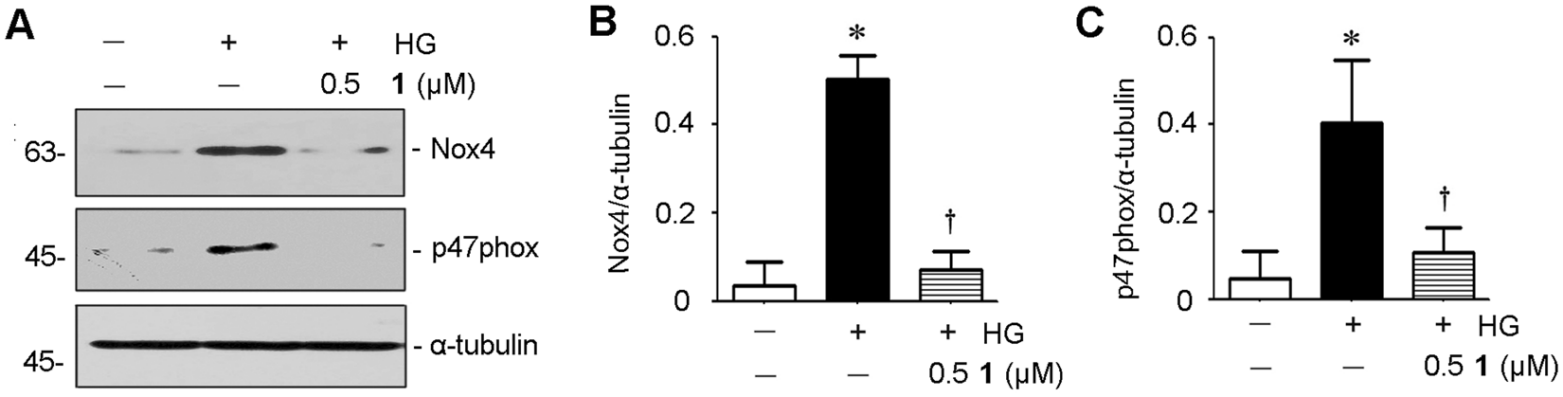
Penicilliumin B (**1**) suppresses oxidative stress in RMC cells. RMC cells were incubated with D-glucose (25 mM) for 24 h in the absence or presence of **1** (0.5 μM). NAPDH oxidase subunits of Nox4 and p47phox were analyzed by western blotting (**A**). Full-length blots are presented in Supporting Information Figure S4. Graphic presentations of Nox4 (**B**) and p47phox (**C**) protein expressions in three groups as indicated. *p < 0.05 vs control (n = 3). ^†^ *p < *0.05 vs HG alone (n = 3).

To further confirm the mechanism of inhibitory effects of **1**, we detected the proliferation and oxidative stress in RMC cells. As shown in Figs 1 and 6, evidently retarded the proliferation of RMC cells as detected by Western blotting analysis of proliferating cell nuclear antigen (PCNA) and c-myc expression. Considering that the inhibition of fibrosis and proliferation in mesangial cells might be associated with the oxidative stress, the NADPH oxidase activation was explored using Western blotting. The results show that upregulation of two key subunits of NAPDH oxidase, Nox4 and p47phox, were indeed inhibited by **1** (Fig. 7), suggesting that **1** exerts antifibrotic and antiproliferative actions in RMC cells through disruption of oxidative stress.

Furthermore, normal epithelial human kidney-2 (HK-2) cells were also used for evaluation of nephrotoxicity *in vitro*. Penicilliumin B (**1**) was showed weak toxic effect on HK-2 cells with IC_50_ value 112.6 μM (Supporting Information, Figure S5).

In conclusion, a novel drimane sesquiterpene methylcyclopentenedione named penicilliumin B (**1**), was obtained from deep sea-derived fungus *Penicillium* sp. F00120, together with three sesquiterpene cyclohexenones (**2**–**4**). This unusual cyclopentenone skeleton seems to be biosynthesized by losing of one carbon from sesquiterpene quinones, which modifies the redox properties of the hydroquinone-quinone functionality and causes significant change in its bioactivities^18^. Penicilliumin B (**1**) was inactive in cytotoxic, antibacterial, antituberculosis, antiviral, and anti-inflammatory assays, however, it was showed to be a promising kidney fibrogenic inhibitor by a mechanism dependent on disruption of oxidative stress in RMC cells, with low toxicity. Although more investigations are needed, our current studies provide proof that penicilliumin B (**1**) can play potential roles in the therapy of diabetic nephropathy, especially on renal fibrotic lesions, presenting a new type of natural product as promising renoprotective agent.

## Methods

### General experimental procedures

UV and IR spectra were measured on an UV-2401PC spectrometer (Shimadzu, Beijing, China) and IR Affinity-1 spectrometer (Shimadzu, Beijing, China), respectively. Optical rotations were performed on a Perkine Elmer 341 polarimeter, and CD spectra were measured with a chirascan circular dichroism spectrometer (Applied Photophysics, Surrey, UK). HR-ESIMS were determined with a Bruker maXis Q-TOF in positive/negative ion mode. The NMR spectra including (1D and 2D NMR) were recorded on a Bruker AC 500 MHz spectrometer using TMS as standard. All chemical shifts were assigned with δ-values. X-ray diffraction intensity data were collected on Agilent Xcalibur Nova single-crystal diffractometer using Cu Kα radiation. Column chromatography (CC) was performed on silica gel (200–300 mesh, 300–400 mesh), and Sephadex LH-20 (Amersham Biosciences, Sweden), respectively. TLC were carried out on silica gel GF254 (10–40 µm) plates (Qingdao Marine Chemical Factory, China). All solvents used were of analytical grade (Tianjin Fuyu Chemical and Industry Factory). Semipreparative HPLC (Agilent Technologies, 1260 infinity series) was performed using an ODS column (YMC-pack ODS-A, 10 × 250 mm, 5 µm, 1.5 mL/min).

### Fungal Material and Fermentation

The fungal strain F00120 was obtained from the sediment of the northern South China Sea at a depth of 1300 m, and identified as we reported before^11^. Seed medium (potato 200 g, dextrose 20 g, NaCl 5 g, distilled water 1000 mL) in 500-mL Erlenmeyer flasks was inoculated with strain F00120 and incubated at 15 °C for 48 h on a rotating shaker (180 rpm). Production medium of solid rice in 1000 mL flasks (rice 200 g, NaCl 4 g, distilled water 200 mL) was inoculated with 10 mL seed solution. Flasks were incubated at 15 °C under static stations and daylight. After 40 days, cultures from 80 flasks were harvested for chemical study.

### Extraction and Isolation

The residue (128 g) was extracted and obtained from the culture medium containing the mycelium, according to the procedures we previously described^11^. Then, it was chromatographed on silica gel and eluted with a gradient of petroleum ether (PE)–EtOAc (1:0, 30:1, 20:1, 10:1, 5:1, 1:1, 0:1) to yield 9 fractions (Frs. A1–A9). Fr. A3 (7.2 g) was further separated by silica gel CC and eluted with PE–MeOH (20:1), resulting in 5 fractions (Frs. D1–D5). Fr. D2 (654.5 mg) was chromatographed on a Sephadex LH-20 column using CHCl_3_−MeOH (1:1) to produce 6 fractions (Frs. F1–F6). Fr. F4 (115.4 mg) was finally purified by semipreparative HPLC eluting with 80% MeOH to give compound **1** (25.5 mg, t_R_ 19.5 min). Compound **2** (43.1 mg) was obtained and purified from Fr. A7, much like we previously described^11^. Compound **3** (8.5 mg, t_R_ 19.0 min) and **4** (12.6 mg, t_R_ 21.3 min) were also isolated from Fr. A7, finally purified by semipreparative HPLC eluting with 75% MeOH.

### New Compound

X-ray Crystallographic Data of **1**. Orthorhombic, C_21_H_30_O_3_; space group *P* 21 21 21 with *a* = 7.35272(11) Å, *b* = 11.03122(14) Å, *c* = 21.7305(3) Å, *V* = 1762.55(4) Å^3^, *Z* = 4, *D*_calcd_ = 1.245 Mg/m^3^, *R*_1_ = 0.0340, *wR*_2_ = 0.0810, and *R* = 0.0318 for *I* > 2*σ*(*I*) data. The absolute configuration was determined on the basis of a Flack parameter of −0.2(4), refined using 5213 Friedel pairs. Crystallographic data for the structure of **1** has been deposited in the Cambridge Crystallographic Data Centre as supplementary publication number 1536108. Copies of these data can be obtained free of charge via www.ccdc.cam.au.ck/conts/retrieving.html (or from the Cambridge Crystallographic Data Centre, 12, Union Road, Cambridge CB21EZ, UK; fax: (t44) 1223-336-033; or deposit@ccd.cam.ac.uk).

*Penicilliumin B (* ***1*** *)*: colorless crystals (MeOH); [α]^25^_D_ + 13.8 (*c* 0.4, MeOH); UV (MeOH) *λ*_max_: 232 nm (Figure S6); IR *ν*_max_: 3354, 3258, 1703, 1635, 667, 596 cm^−1^ (Figure S7); CD (0.5 mM, MeOH) *λ*_max_ (*Δ*ε) 234 (+16.10), 215 (+8.84) (Figure S8); ^1^H and^13^C NMR data, see Table 1; HR-ESI-MS (*m/z* 353.2114 [M + Na]^+^, calcd for C_21_H_30_O_3_Na, 353.2087; *m/z* 331.2267 [M + H]^+^, calcd for C_21_H_31_O_3_, 331.2268) (Figure S9).

### Renoprotective assay *in vitro*

Rat glomerular mesangial cells (RMC, HBZY-1, Life-Science Academy of Wuhan University, Wuhan, China) were cultured and maintained in DMEM (Invitrogen, Carlsbad, CA), PH7.4, supplemented with 10% fetal bovine serum (FBS, Invitrogen, Carlsbad, CA), 2 mM glutamine, 100 U/ml penicillin, 100 µg/ml streptomycin at 37 °C. To examine the effect of compound **1**, RMC cells were pre-treated with indicated concentration of compound **1** at 37 °C for 1 h, and then exposed to either 5.6 mM (normal glucose, NG) or 25 mM (high glucose, HG) D-glucose for 24 h or 48 h. Whole cell proteins were extracted using a cell lysis buffer kit (Cell signaling, MA, USA) and quantified by the Bradford assay (Bio-Rad, Hercules, CA). The equivalent amount of proteins were resolved with SDS-PAGE and transferred to nitrocellulose membranes. The membranes were blocked and then incubating with a rabbit anti-fibronectin pAb (Sigma, MO, USA), rabbit anti-collagen I (Abcam, MA, USA), rabbit anti-p47phox, rabbit anti-Nox4, rabbit anti-PCNA, rabbit anti-c-myc (all from Santa Cruz, Texas, USA) and mouse anti-α-tubulin (Sigma) at 4 °C overnight. After washing, the membranes were incubated with HRP-conjugated anti-rabbit or anti-mouse IgG secondary antibodies and detected by using ECL detection system.

### Toxic effect on normal epithelial human kidney-2 (HK-2) cells

HK-2, an immortalized proximal tubular cell line derived from normal adult human kidney was obtained from the American Type Culture Collection (ATCC; Manassas, VA, USA), and were grown in Dulbecco’s Modified Eagle Media/F12 (D/F12) medium supplemented with 10% FBS in a humidified incubator at 37 °C in the presence of 5% CO_2_. The culture medium was changed every 2 days. HK-2 cell viability was evaluated by the CCK-8 assay. Briefly, HK-2 cells (1 × 10^4^ cells/well) were seeded in 96-well microplates, and then cultured in D/F12 growth medium for 24 h. Subsequently, the medium was replaced with D/F12 growth medium containing different concentrations compound **1** (0.05, 0.5, 4.0, 20.0, 80.0, 160.0, and 320.0 μM). After 48 h, 10 μl of the CCK-8 assay solution was added into each well, followed by incubation of the microplates at 37 °C in 5% CO_2_/95% air for 2 h. Finally, absorption was measured at 450 nm using a microplate reader (PerkinElmer, Inc., Waltham, MA, USA), with a reference wavelength of 650 nm.

### Supporting Information

The bioassay (cytotoxic against cancer cells, antibacterial, antituberculosis, antiviral, and antiinflammatory) data of **1**; UV, IR, CD, ^1^H, ^13^C and 2 D NMR spectra of **1** .

## Electronic supplementary material

Supplementary Information
