# Supplementary material for: Penicilliumin B, a novel sesquiterpene methylcyclopentenedione from a deep sea-derived Penicillium strain with renoprotective activities
Source: Sci Rep. 2017 Sep 7;7:10757. doi: 10.1038/s41598-017-11007-4 (PMC5589727; doi:10.1038/s41598-017-11007-4)
Supplement: Supplementary file 1 — Supplementary Information [file 41598_2017_11007_MOESM1_ESM.pdf]

Supporting Information

Penicilliumin B, a novel sesquiterpene methylcyclopentenedione from a deep sea-derived *Penicillium* strain with renoprotective activities

Xiuping Lin[1,#], Qinyu Wu[2,3,#], Yuying Yu[1], Zhi Liang[2], Yonghong Liu[1,*], Lili Zhou[3,*], Lan Tang[2,*], & Xuefeng Zhou[1,*]

[1] Chinese Academy of Sciences (CAS) Key Laboratory of Tropical Marine Bio-resources and Ecology, Guangdong Key Laboratory of Marine Materia Medica, South China Sea Institute of Oceanology, CAS, Guangzhou 510301, China

[2] Biopharmaceutics, Guangdong Provincial Key Laboratory of New Drug Screening, School of Pharmaceutical Sciences, Southern Medical University, Guangzhou 510515, China

[3] State Key Laboratory of Organ Failure Research, National Clinical Research Center of Kidney Disease, Division of Nephrology, Nanfang Hospital, Southern Medical University, Guangzhou 510515, China

[#] These authors contributed equally.

* Correspondence and requests for materials should be addressed to Y.L. (email: yonghongliu@scsio.ac.cn), or L.Z. (email: jinli730@smu.edu.cn), or L.T. (email: tl405@smu.edu.cn), or X.Z. (email: xfzhou@scsio.ac.cn).

Contents

Bioassay Experimental Section

(1) Cytotoxicity assay

(2) Antibacterial and antituberculosis assay

(3) Antiviral assay

(4) Anti-inflammatory assay

Supplemental references

Table S1. Inhibition rate (%) of **1** (30 μM) against eight human cancer cells.

Table S2. Inhibition rate (%) of **1** (30 μM) against three common bacteria and a tubercle bacillus.

Table S3. Inhibition rate (%) of **1** (30 μM) against H1N1, H3N2 and EV71 virus.

Table S4. Inhibition rate (%) of **1** (30 μM) against COX-1 and COX-2.

Figure S1. NF-κB inhibition activities of **1**.

Figure S2. Full unedited gels for Figure 5.

Figure S3. Full unedited gels for Figure 6.

Figure S4. Full unedited gels for Figure 7.

Figure S5. Effect of **1** on HK-2 cell viability.

Figure S6. UV spectrum of Penicilliumin B (**1**).

Figure S7. IR spectrum of Penicilliumin B (**1**).

Figure S8. CD spectrum of Penicilliumin B (**1**).

Figure S9. HR-ESI-MS spectrum of **1.**

Figure S10. ^{1}H-NMR (500 MHz) spectrum of Penicilliumin B (**1**).

Figure S11. ^{13}C-NMR (500 MHz) spectrum of Penicilliumin B (**1**).

Figure S12. DEPT (500 MHz) spectrum of Penicilliumin B (**1**).

Figure S13. HSQC (500 MHz) spectrum of Penicilliumin B (**1**).

Figure S14. HMBC (500 MHz) spectrum of Penicilliumin B (**1**).

Figure S15. ^{1}H-^{1}H COSY (500 MHz) spectrum of Penicilliumin B (**1**).

Figure S16. NOESY (500 MHz) spectrum of Penicilliumin B (**1**).

Bioassay Experimental Section

(1) Cytotoxicity assay

Eight human cancer cell lines (K562, MCF-7, Hela, Du145, H1975, A549, MOLT-4, and HL60) were purchased from Shanghai Cell Bank, Chinese Academy of Sciences. The cytotoxic activities against those cells were determined according to previously reported methods [S1,S2]. If the inhibitory rate is exceed 30% in preliminary test with the concentration of 30 μM, various concentrations of the compounds will be used and measured again, to calculate the IC_{50} values.

(2) Antibacterial and antituberculosis assay

Antibacterial assay. Antibacterial against *Escherichia coli, Salmonella enterica*, and *Staphylococcus aureus* were carried out using discagar diffusion method [S3]. If the inhibitory rate is exceed 30% in preliminary test with the concentration of 30 μM, various concentrations of the compounds will be used and measured again, to calculate the IC_{50} values.

Antituberculosis assay. Inhibition rate of compounds against a tubercke bacillus was proceeded. Autoluminescent *Mycobacterium tuberculosis* H37Ra was inoculated in a 50 mL centrifuge tube containing 5 mL of 7H9 broth (Becton Dickinson) with 0.1% Tween 80 and 10% OADC enrichment (Becton Dickinson) and then incubated at 37 °C. When the cultures reached an OD_{600} nm of 0.3 to 1.0, the culture was diluted, and 50 μL diluted H37Ra was inoculated in sterile 384 well plates, the RLU of which should be between 10 000 and 50 000 and should be recorded as the base luminescent Day0. The compounds were added to the 384 well plates in triplicate with a preliminary concentration of 30 μM. Rifampicin was used as positive drug. The luminescent value was detected for the following 3 d. [S2]

(3) Antiviral assay

The antiviral activities against H1N1 and H3N2 were evaluated by the cytopathic effect (CPE) inhibition assay, on Madin–Darby canine kidney (MDCK) cells with CCK-8 assay, according to previously reported methods [S2, S4]. And the antiviral bioassay against EV71 virus was measured the ability to inhibit the CPE induced by EV71 virus on Vero cells with CCK-8 assay, according to previously reported methods [S2, S4]. If the inhibitory rate is exceed 30% in preliminary test with the concentration of 30 μM, various concentrations of the compounds will be used and measured again, to calculate the IC_{50} values.

(4) Anti-inflammatory assay

The compound **1** was tested for COX-1 and COX-2 inhibitory activities using the COX (ovine) Inhibitor Screening Kits according to the manufacturer's instructions, as previously described [S5, S6]. Concentration of 30 μM of **1** was used in the preliminary tests.

To examine NF-κB inhibition activities, RAW264.7 cells stably transfected with a luciferase reporter gene was used. Cells were plated in 96-well plates at adensity of 1×10^4 cells/well and pre-treated with different concentrations compound **1** (0, 3.125, 6.25, 12.5, 25, 50, and 100 μM) for 30 min, followed by 1 μg/ml LPS stimulation for 6h. BAY11-7082, obtained from Sigma-Aldrich (St Louis, MO, USA), was used as positive control (10 μM). Cells were harvested and luciferase activity was measured using the luciferase assay system (Promega, Madison, WI) [S7].

Supplemental references

[S1] Lin, X.; Zhou, X.; Wang, F.; Liu, K.; Yang, B.; Yang, X.; Peng, Y.; Liu, J.; Ren, Z.; Liu, Y. *Mar. Drugs* **2012**, *10*, 106–115.

[S2] Fang, W.; Lin, X.; Zhou, X.; Wan, J.; Lu, X.; Yang, B.; Ai, W.; Lin, J.; Zhang, T.; Tu, Z.; Liu, Y. *Med. Chem. Comm*. **2014**, 5, 701–705.

[S3] Ai, W.; Lin, X.; Wang, Z.; Lu, X.; Mangaladoss, F.; Yang, X.; Zhou, X.; Tu, Z.; Liu, Y. *J. Antibiotic.* **2014**, 68, 213–215.

[S4] Zeng, Y.-B.; Wang, H.; Zuo, W.-J.; Zheng, B.; Yang, T.; Dai, H.-F.; Mei, W.-L. *Mar. Drugs* **2012**, *10*, 598–603.

[S5] Fang, W.; Lin, X.; Wang, J.; Liu, Y.; Tao, H.; Zhou, X. *Molecules.* **2016,** *21*, 941.

[S6] Ai, W.; Lin, X.P.; Tu, Z.; Tian, X.P.; Lu, X.; Mangaladoss, F.; Zhong, Z.L.; Liu, Y. *Nat. Prod. Res*. **2014**, 28, 1219–1224.

[S7] Yang, B.; Wei, X.; Huang, J.; Lin, X.; Liu, J.; Liao, S.; Wang, J.; Zhou, X.; Wang L.; Liu, Y. *Mar. Drugs.* **2014**, *12*, 5316–5327.

Table S1. Inhibition rate (%) of **1** (30 μM) against eight human cancer cells.

Cells	K562	MCF-7	Hela	Du145	H1975	A549	MOLT-4	HL60
Inhibition rate (%)	15.4	13.0	29.8	–4.0	8.0	5.0	14.7	–13.5

Table S2. Inhibition rate (%) of **1** (30 μM) against three common bacteria and a tubercle bacillus.

Bacteria	*Escherichia coli*	*Salmonella enterica*	*Staphylococcus aureus*	*Mycobacterium tuberculosis* H37Ra
Inhibition rate (%)	2.61	–15.51	17.61	16.39

Table S3. Inhibition rate (%) of **1** (30 μM) against H1N1, H3N2 and EV71 virus.

Virus	H1N1	H3N2	EV71
Inhibition rate (%)	–17.37	5.76	–1.69

Table S4. Inhibition rate (%) of **1** (30 μM) against COX-1 and COX-2.

Targets	COX-1	COX-2
Inhibition rate	–18.30	–16.37

.

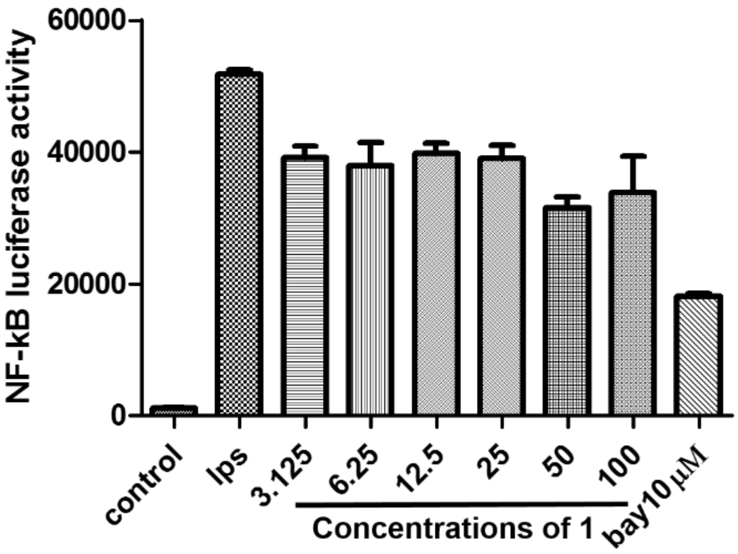

Figure S1. NF-κB inhibition activities of **1**.

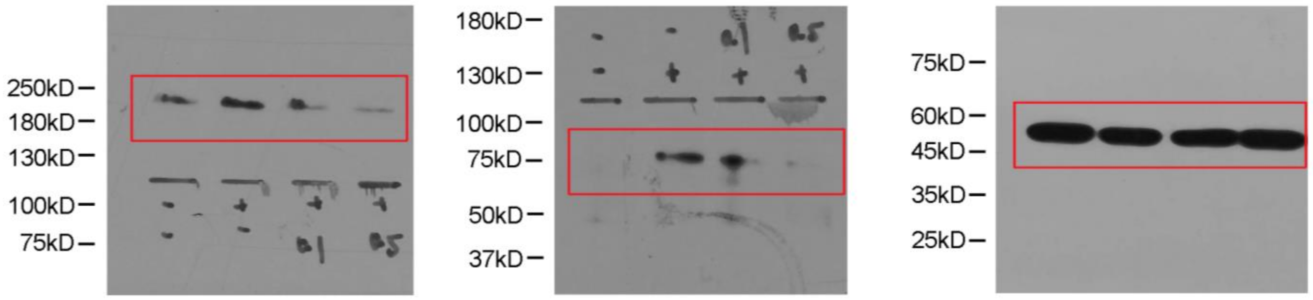

Figure S2. Full unedited gels for Figure 5.

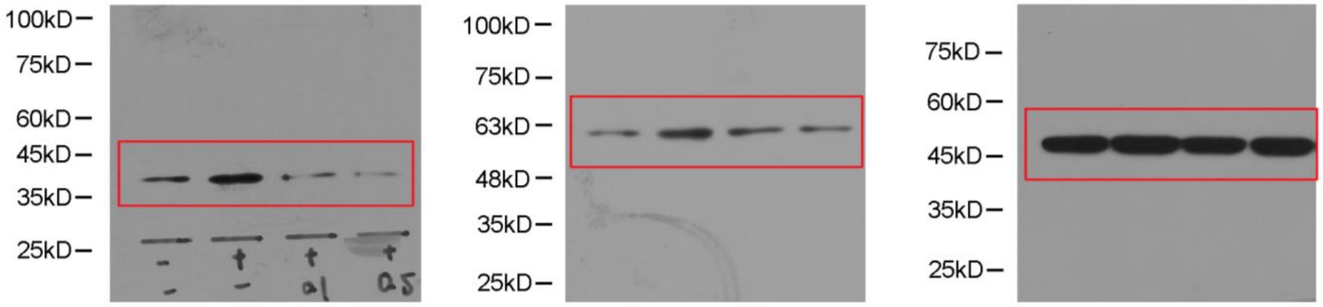

Figure S3. Full unedited gels for Figure 6.

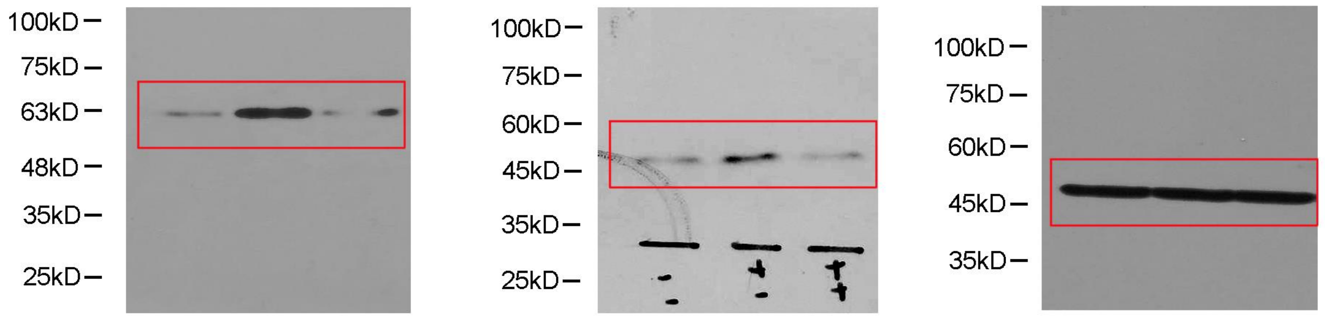

Figure S4. Full unedited gels for Figure 7.

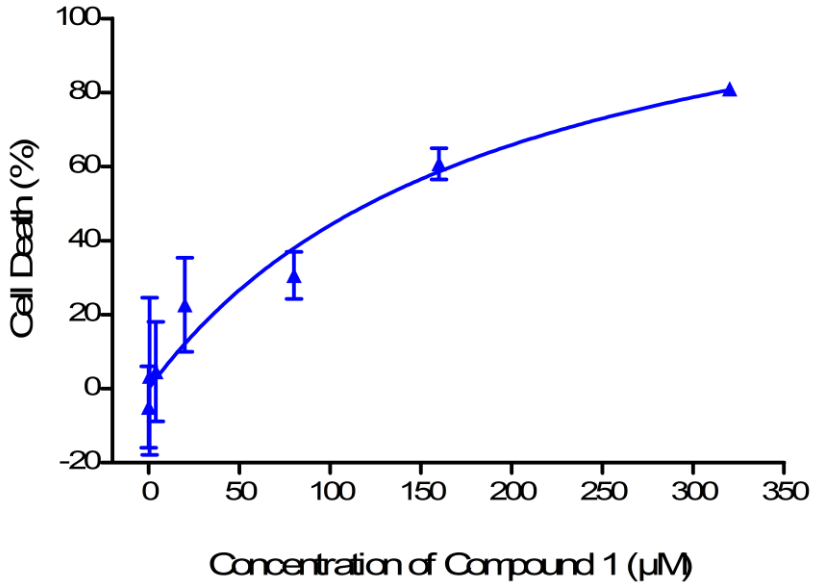

Figure S5. Effect of **1** on HK-2 cell viability.

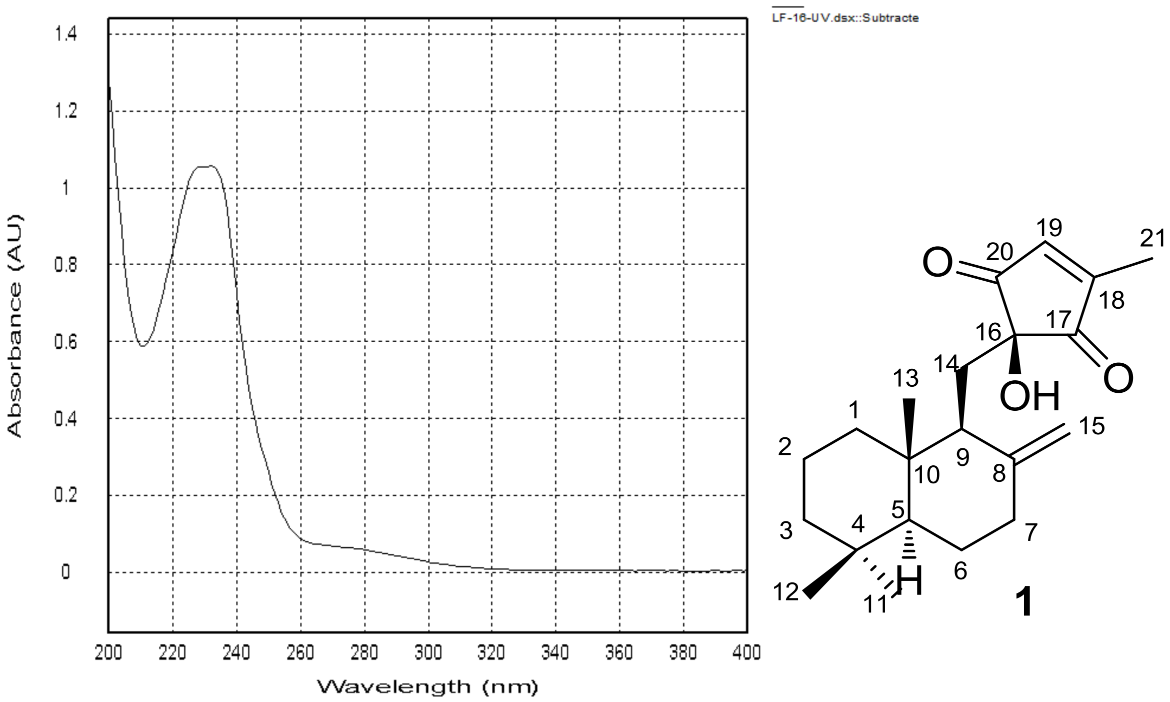

Figure S6. UV spectrum of Penicilliumin B (**1**).

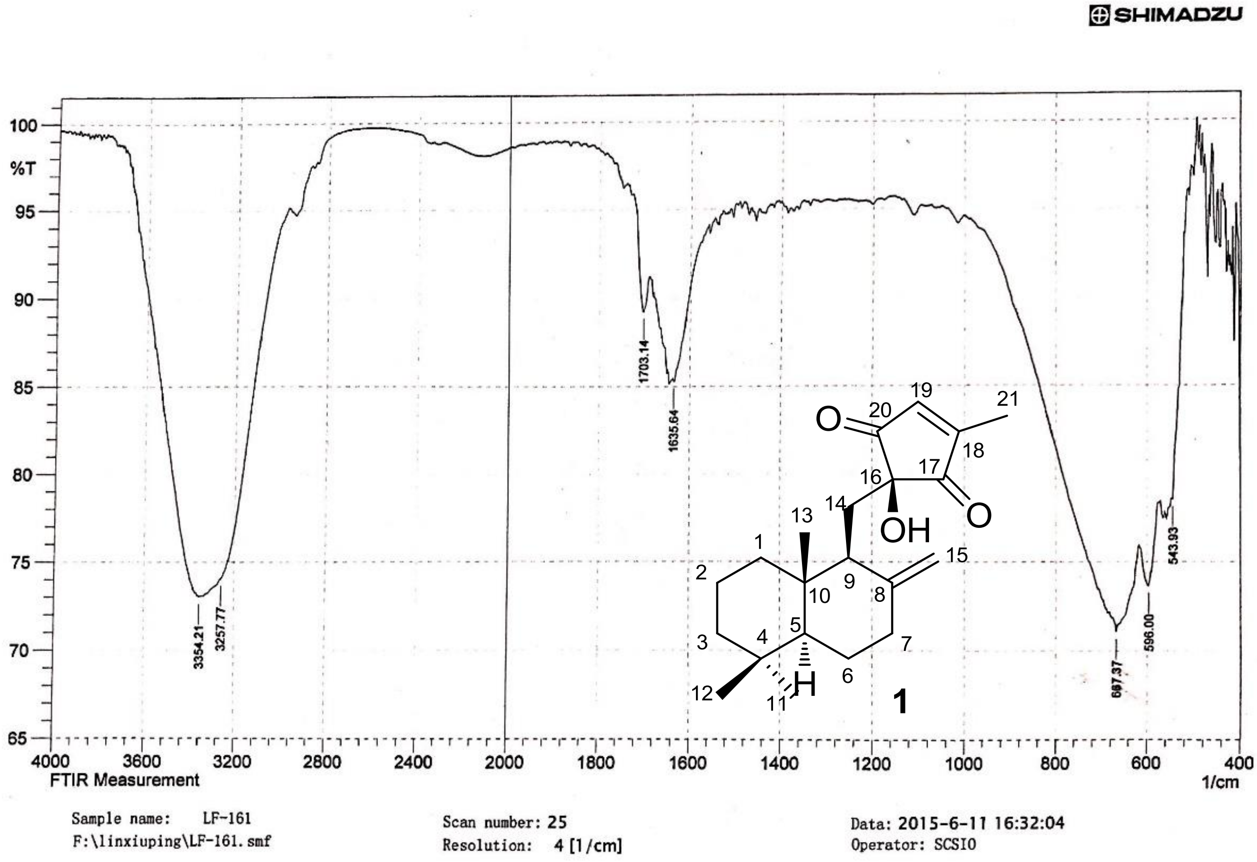

Figure S7. IR spectrum of Penicilliumin B (**1**).

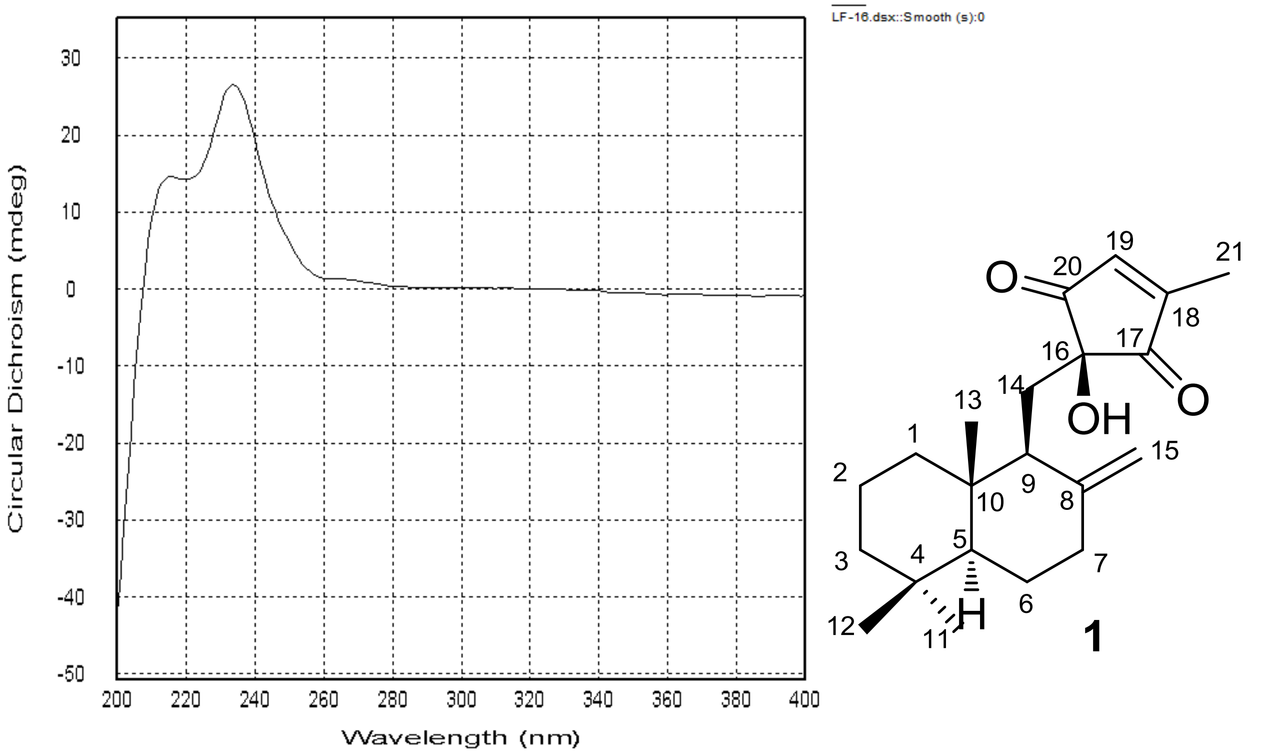

Figure S8. CD spectrum of Penicilliumin B (**1**).

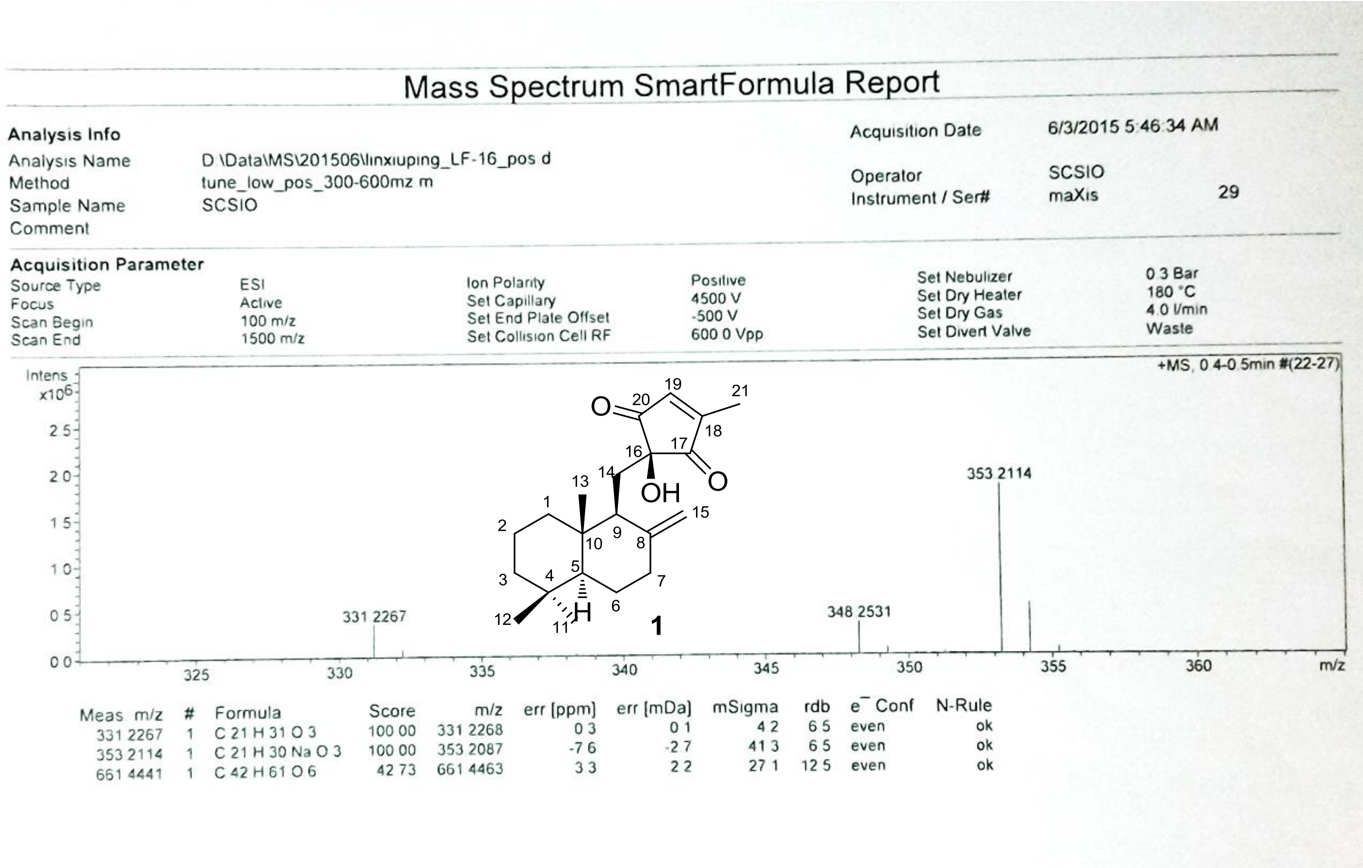
Mass Spectrum SmartFormula Report

Analysis Info

Analysis Name D \Data\MS\201506\linxiuping_LF-16_pos d
Method tune_low_pos_300-600mz m
Sample Name SCSIO
Comment

Acquisition Date 6/3/2015 5:46:34 AM
Operator SCSIO
Instrument / Ser# maXis 29

Acquisition Parameter

Source Type ESI
Focus Active
Scan Begin 100 m/z
Scan End 1500 m/z
Ion Polarity Positive
Set Capillary 4500 V
Set End Plate Offset -500 V
Set Collision Cell RF 600 0 Vpp
Set Nebulizer 0 3 Bar
Set Dry Heater 180 °C
Set Dry Gas 4.0 l/min
Set Divert Valve Waste

Meas. m/z	#	Formula	Score	m/z	err [ppm]	err [mDa]	mSigma	rdb	e⁻ Conf	N-Rule
331 2267	1	C 21 H 31 O 3	100 00	331 2268	0 3	0 1	4 2	6 5	even	ok
353 2114	1	C 21 H 30 Na O 3	100 00	353 2087	-7 6	-2 7	41 3	6 5	even	ok
661 4441	1	C 42 H 61 O 6	42 73	661 4463	3 3	2 2	27 1	12 5	even	ok

Figure S9. HR-ESI-MS spectrum of Penicilliumin B (**1**).

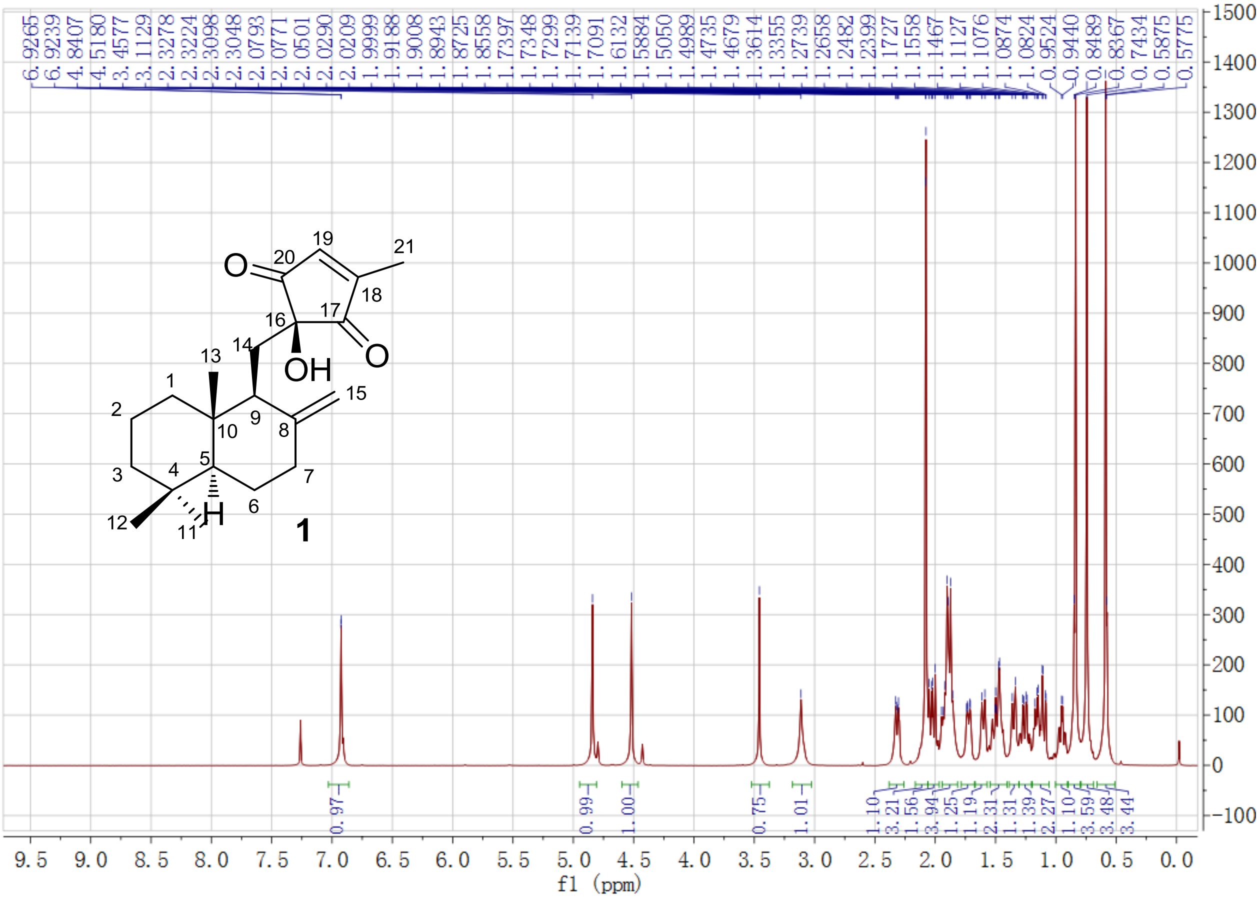

Figure S10. ^{1}H-NMR (500 MHz) spectrum of Penicilliumin B (**1**).

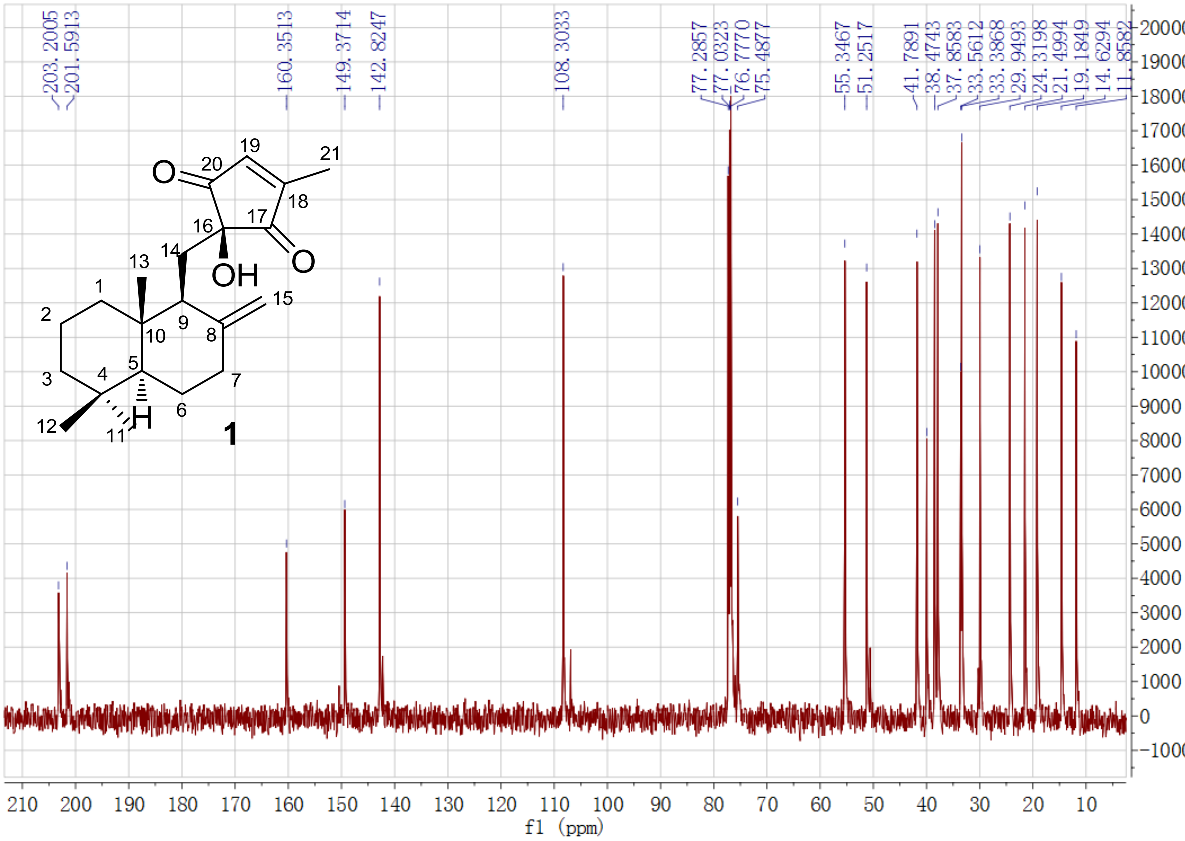

Figure S11. ^{13}C-NMR (500 MHz) spectrum of Penicilliumin B (**1**).

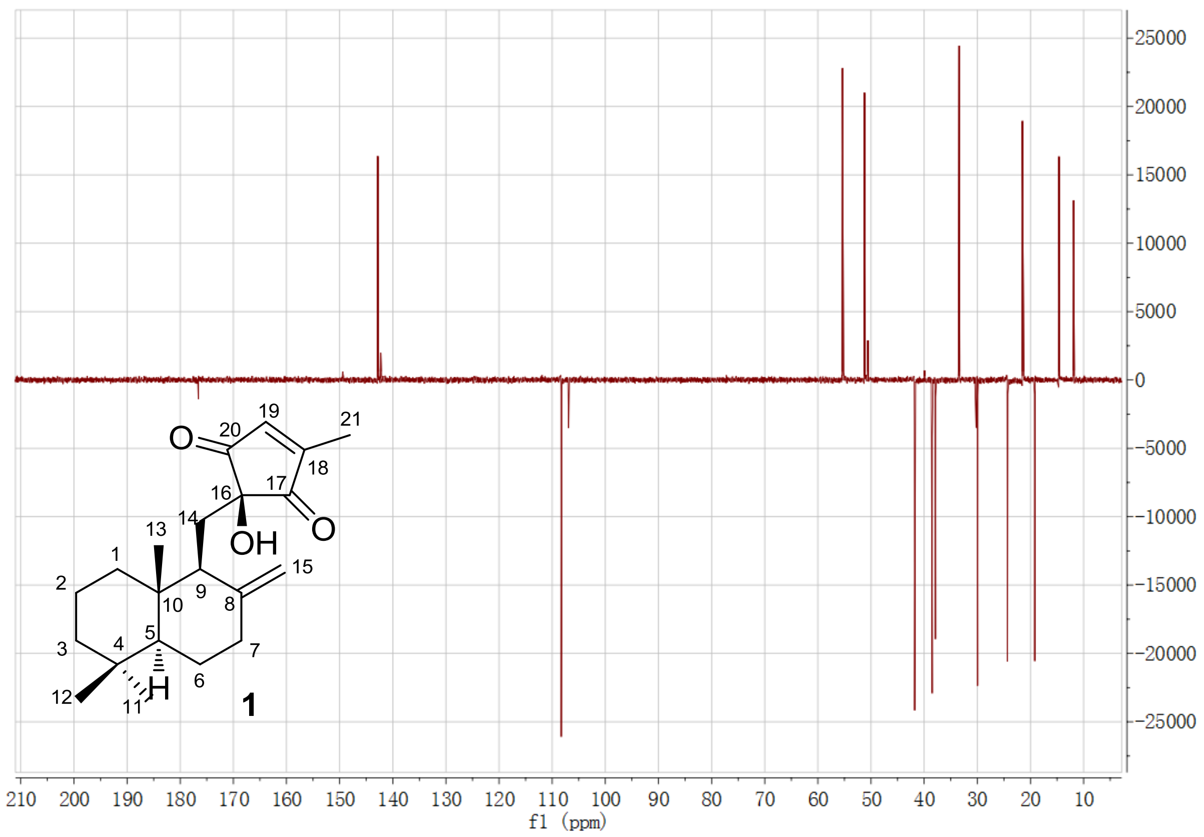

Figure S12. DEPT (500 MHz) spectrum of Penicilliumin B (**1**).

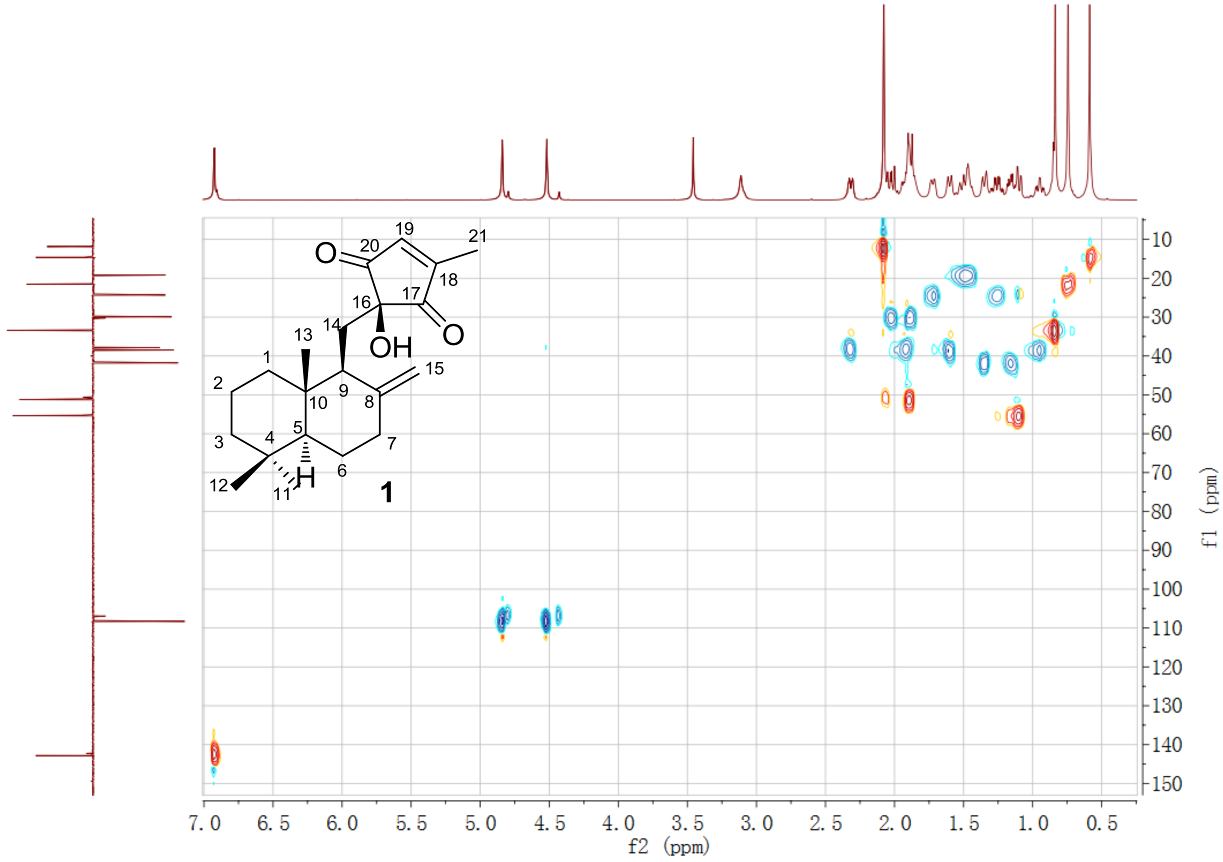

Figure S13. HSQC (500 MHz) spectrum of Penicilliumin B (**1**).

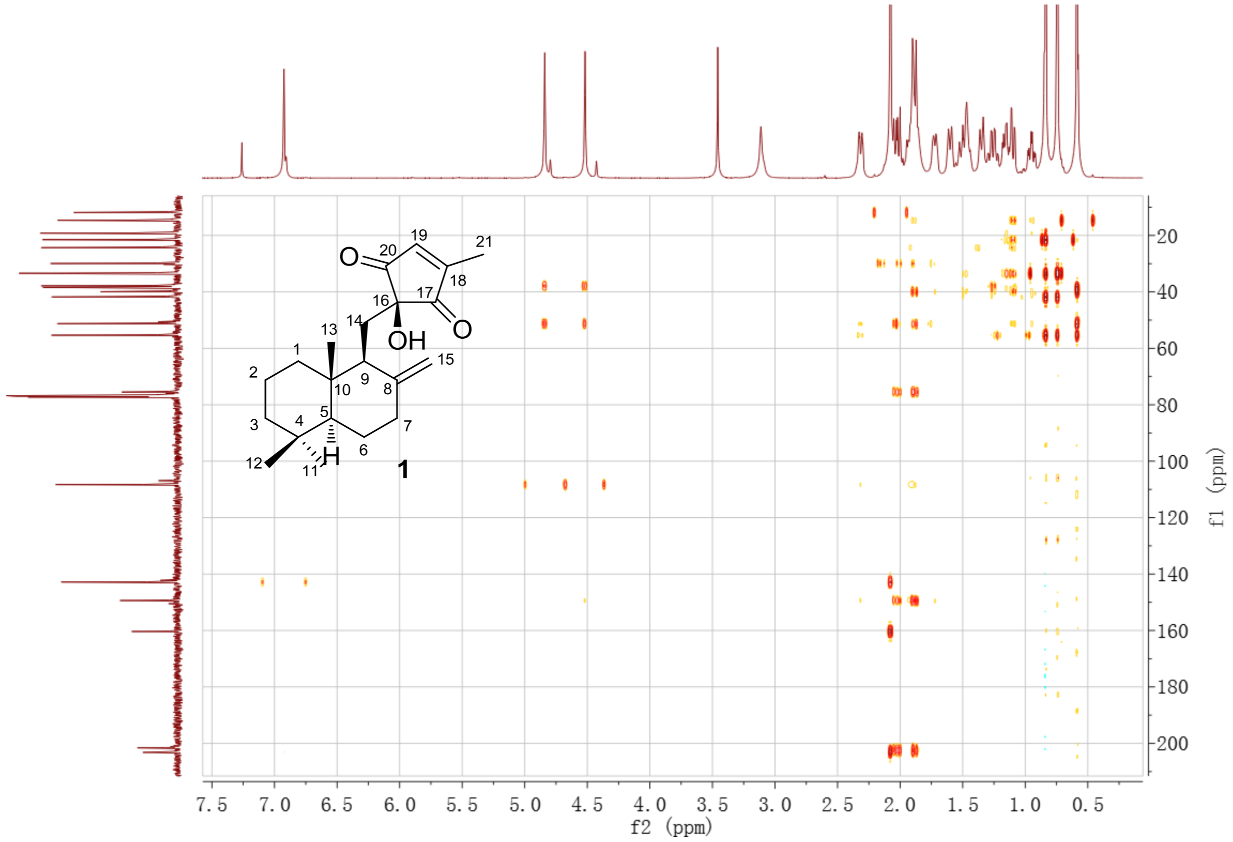

Figure S14. HMBC (500 MHz) spectrum of Penicilliumin B (**1**).

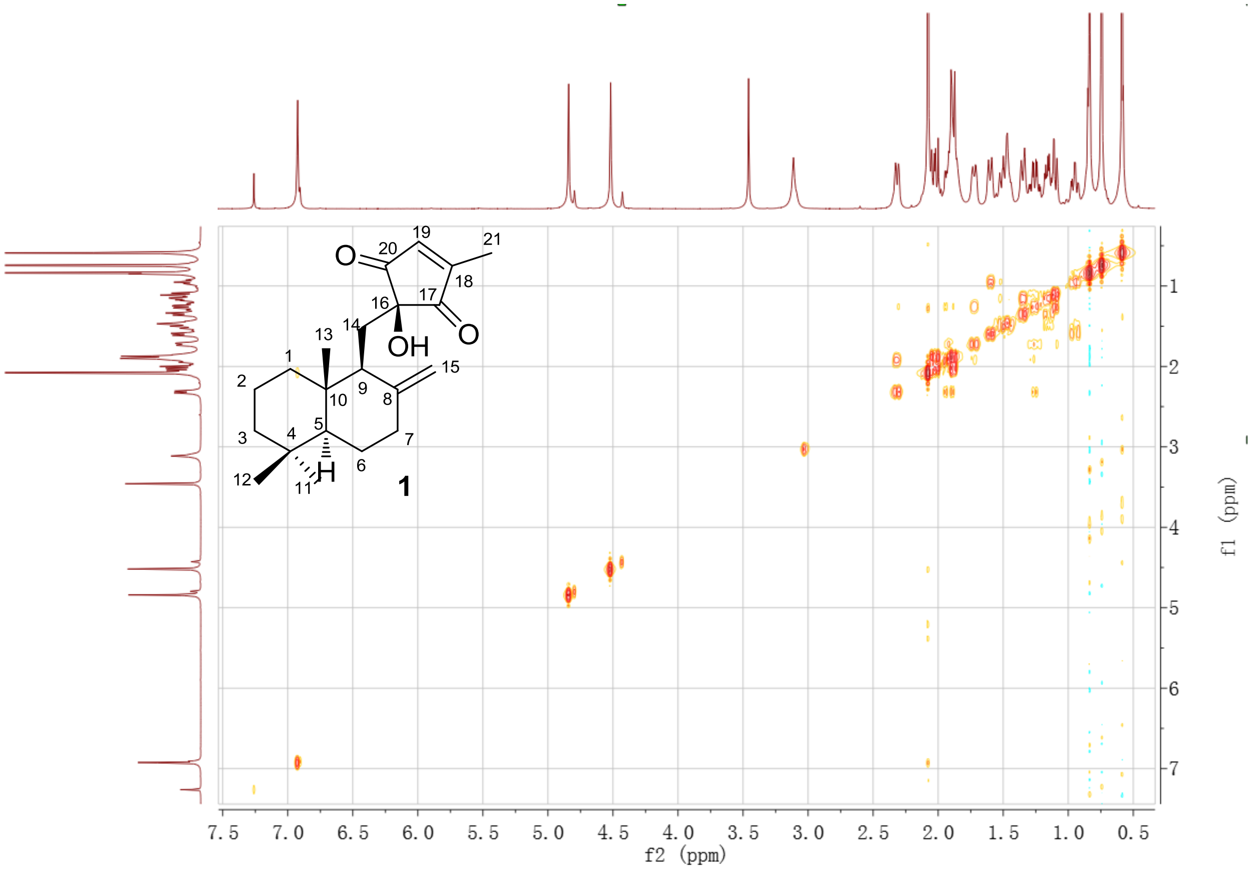

Figure S15. ^{1}H-^{1}H COSY (500 MHz) spectrum of Penicilliumin B (**1**).

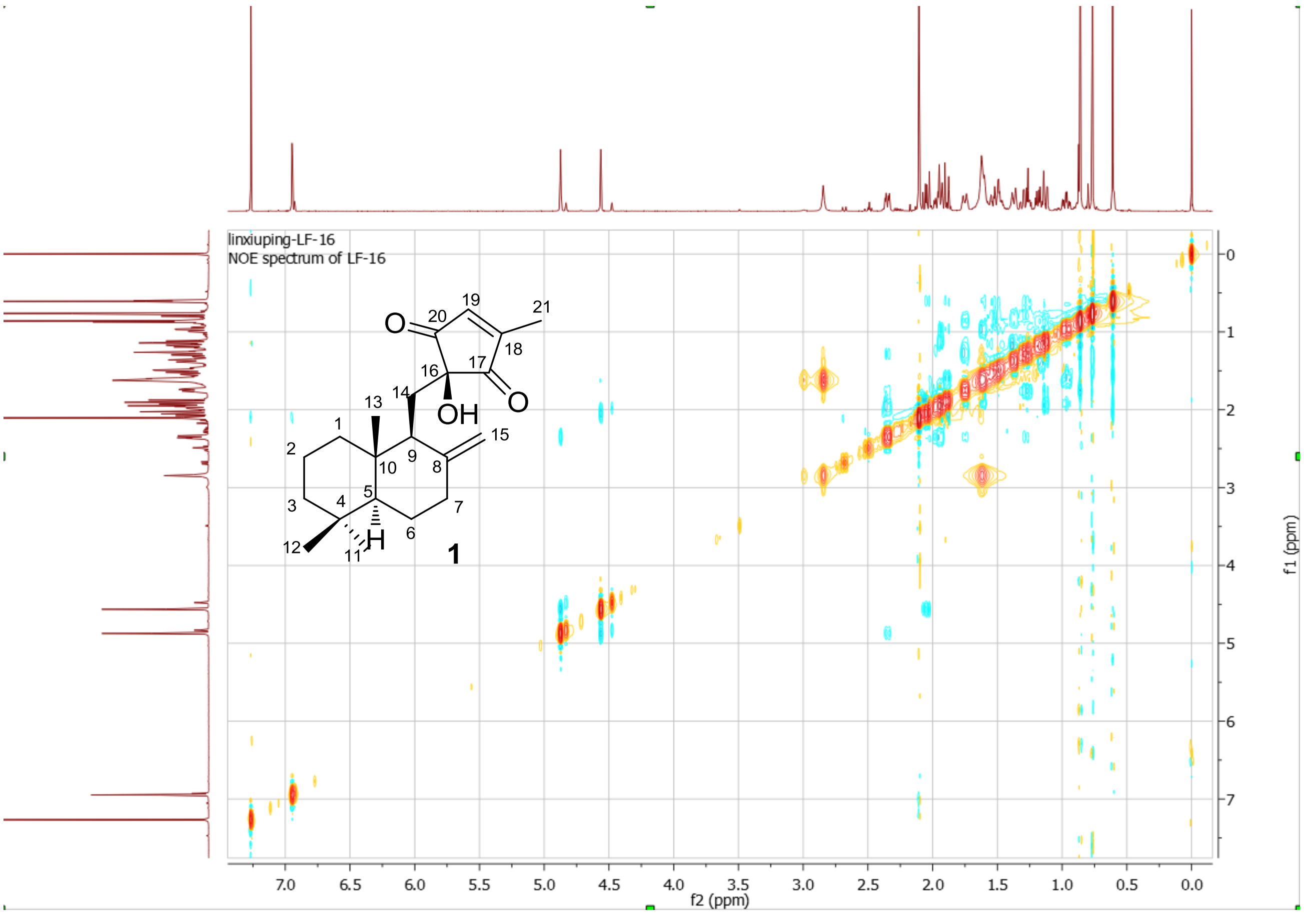

Figure S16. NOESY (500 MHz) spectrum of Penicilliumin B (**1**).
